# BrMYB116 transcription factor enhances Cd stress tolerance by activating FIT3 in yeast and Chinese cabbage

**DOI:** 10.3389/fpls.2024.1388924

**Published:** 2024-06-07

**Authors:** Ali Anwar, Chao Yuan, Bing Cui, Lixia Wang, Lilong He, Jianwei Gao

**Affiliations:** ^1^ Institute of Vegetables, Shandong Key Laboratory of Greenhouse Vegetable Biology, Shandong Branch of National Vegetable Improvement Center, Huanghuai Region Vegetable Scientific Station of Ministry of Agriculture (Shandong), Shandong Academy of Agricultural Sciences, Jinan, China; ^2^ College of Horticulture, South China Agriculture University, Guangzhou, China; ^3^ Key Laboratory of Plant Development and Environment Adaptation Biology, Ministry of Education; School of Life Science, Shandong University, Qingdao, China

**Keywords:** Chinese cabbage, abiotic stress, BrMYB116, FIT3, Cd stress, RNA-seq

## Abstract

Cd (cadmium) is a highly toxic heavy metal pollutant often present in soil and detrimentally impacting the production and quality of horticultural crops. Cd affects various physiological and biochemical processes in plants, including chlorophyll synthesis, photosynthesis, mineral uptake and accumulation, and hormonal imbalance, leading to cell death. The MYB family of transcription factors plays a significant role in plant response to environmental influences. However, the role of MYB116 in abiotic stress tolerance remains unclear. In this study, we reported that Chinese cabbage transcription factor BrMYB116 enhanced Cd stress tolerance in yeast. The expression level of BrMYB116 was increased by Cd stress in Chinese cabbage. Additionally, yeast cells overexpressing BrMYB116 showed improved Cd stress tolerance and reduced Cd accumulation. Moreover, we found that BrMYB116 interacted with facilitator of iron transport (FIT3) to enhance Cd stress tolerance. ChIP-qPCR results showed that ScFIT3 was activated through specific binding to its promoter. Additionally, the overexpression of ScFIT3 induced Cd stress tolerance and reduced Cd accumulation in yeast and Chinese cabbage. These results suggest new avenues for plant genomic modification to mitigate Cd toxicity and enhance the safety of vegetable production.

## 1 Introduction

Over the last two decades, cadmium (Cd) has emerged as a severe environmental threat to the plant kingdom and human health. Cd is one of the most highly toxic heavy metals, easily translocated in soil and absorbed by various plants, such as rice, Chinese cabbage, and carrot (Yang et al., 2009; Yang et al., 2016; Bashir et al., 2018). Cd has been linked to a variety of human health issues, including cancer and cardiovascular diseases (Lee et al., 2005; Jarup and Akesson, 2009; Melila et al., 2019). The rapid increase in industrialization and improper application of pesticides and chemical fertilizers have led to widespread soil contamination by Cd affecting not only the plants but also humans through the food chain. Cd accumulation in cells or tissues can inhibit cell growth via a wide range of physiological, biochemical, morphological, cellular, and ultrastructural changes (Bertin and Averbeck, 2006; Parrotta et al., 2015; Yang et al., 2018; Jin et al., 2019; Chen et al., 2020). Cd promotes the accumulation of reactive oxygen species (ROS) and malondialdehyde (MDA), which are also highly toxic and trigger various cellular alterations at the cellular level (Li et al., 2015; Cui et al., 2017; Kamran et al., 2019; Zhang et al., 2020). At the molecular level, extensive research has been conducted on Cd stress revealing many candidate genes that encode metal transporters and transcription factor family genes that regulate Cd detoxification and enhance tolerance in plants and other species (Ueno et al., 2010; Gallego et al., 2012; Sasaki et al., 2012; He et al., 2016; Cai et al., 2017).

The MYB proteins belong to a superfamily of plant transcription factors (TFs), which play a central role in regulating abiotic responses. MYB transcription factor family genes are involved in plant growth, developmental processes, and abiotic stress tolerance (Baldoni et al., 2015; Liu et al., 2015). MYB family genes are classified into four subfamilies, 1R, 2R, 3R, and 4R-MYB (Jin and Martin, 1999; Katiyar et al., 2012), each featuring one or more conserved structural domains. 2R-MYB is the most prevalent type in plants (Katiyar et al., 2012; Salih et al., 2016). Thousands of MYB family proteins have been identified to regulate physiological, biochemical, and molecular processes under normal and abiotic stresses (Dubos et al., 2010; Liu et al., 2017; Wang et al., 2021). A number of studies have reported on MYB genes conferring stress tolerance in plants. For example, *MYBS1* induces drought stress tolerance in *Arabidopsis*, *PsnMYB108* enhances salinity stress in tobacco, and *RmMYB108* enhances cold stress in *Arabidopsis* (Lin et al., 2014; Zhao et al., 2020; Dong et al., 2021). MYB15 regulates cold stress tolerance by interacting with the inducer of C-repeat-binding factor (CBF) expression 1 (ICE1) and binding to the promoter of CBF (Kim et al., 2017). The overexpression of BnMYB2 in *Boehmeria nivea* enhances Cd stress tolerance and reduces the accumulation in *Arabidopsis* (Zhu et al., 2020). Similarly, OsMYB45 plays an essential role in resistance to Cd stress in rice (Hu et al., 2017). SbMYB15 from *Salicornia brachiata* (Roxb) alleviates Cd and nickel stress in transgenic tobacco by limiting uptake and modulating antioxidative defense systems (Sapara et al., 2019).

Numerous MYB family genes have been identified in the Brassicaceae family to regulate plant response to environmental cues. For example, BcMYB111 induces flavonol biosynthesis to enhance cold tolerance under stress in non-heading Chinese cabbage (Chen et al., 2023). *AtMYB20* confers drought tolerance in *Arabidopsis* (Gao et al., 2014). The R2R3–MYB transcription factor AtMYB49 positively regulates Cd accumulation via associating with the basic-look-helix–look–helix transcription factors bHLH38 and bHLH101, which activate iron-regulated transporter 1 (IRT1) and heavy-metal-associated isoprenylated proteins (HIPP22 and HIPP44) (Zhang et al., 2019). In addition, AtMYB4 regulates Cd tolerance by providing enhanced protection against oxidative damage and upregulating the expression of PCS1 and MT1C in *Arabidopsis* (Agarwal et al., 2020). However, most identified MYB transcription factor family genes did not regulate Cd stress tolerance with the exception of BnMYB2 and AtMYB49.

Chinese cabbage (*Brassica rapa* subsp. *pekinensis*) is an essential leafy vegetable in East Asia. Like many other leafy vegetables, it exhibits a high capacity for accumulating Cd in its leaves (Kuboi et al., 1986; Huang et al., 2017; Rizwan et al., 2017). Therefore, it is essential to identify Cd tolerance candidate genes in Chinese cabbage. In this study, transcript profiling analyses were performed to identify these genes in Chinese cabbage using the “Guangdongzao” cultivar (He et al., 2022). Notably, BrMYB116, an MYB transcription factor in Chinese cabbage, has been discovered to enhance Cd tolerance, as demonstrated in a yeast system. Previously, MYB116 from sweet potatoes has been demonstrated to play an important role in drought tolerance (Zhou et al., 2019). However, the downstream target genes have not been identified. For further understanding, we generated BrMYB116-overexpressing line in Chinese cabbage. Surprisingly, these transgenic plants did not show enhanced Cd tolerance compared with the wild-type control, which differs from the yeast phenotype. Consequently, we performed RNA-seq transcriptome analysis on Cd-treated yeast cells carrying either an empty vector (WT) or BrMYB16 (overexpression), through which, 18 differentially expressed genes (DEGs) were identified. Notably, DEG5 (FIT3, CENPK1137D_2397), the only overexpressing DEG in yeast, greatly induces Cd stress tolerance and reduces Cd accumulation within the yeast cells mirroring the Cd tolerance phenotype displayed by BrMYB116. More importantly, FIT3 can enhance Cd tolerance and reduce Cd accumulation when overexpressed in Chinese cabbage. Given the absence of FIT3 homologs in Chinese cabbage, the loss of MYB116 in Chinese cabbages relative to Cd tolerance in these plants may be attributed to the evolutionary loss of FIT3.

Our results further demonstrated that the MYB116 protein directly binds to the FIT3 promoter, thereby activating FIT3 in response to Cd stress. We hypothesize that *FIT3* acts as a downstream gene to mediate the iron transporter channel in Cd stress tolerance and Cd exclusion. This study illuminates a previously unexplored molecular foundation underlying tolerance to Cd stress, thereby laying a foundational pathway for future investigations on the development of genetically modified crops with enhanced Cd stress tolerance.

## 2 Materials and methods

### 2.1 Plant treatments

The seeds of Chinese cabbage (Cv. Guangdongzao) were pretreated by soaking them with 8% sodium hypochlorite for 3 min, followed by thorough washing with ddH_2_O at least five times. These seeds were then germinated in half-strength MS media within a growth chamber set to day/night cycles of 16 h/8 h at 22°C/20°C. Seedlings with uniform size were transferred to a hydroponic culture. After 5 days, they were subjected to a 75-μM Cd treatment. This concentration was selected as it yielded the most pronounced tolerance phenotype in BrMYB116 transgenic yeast compared to the control, a finding supported by testing other concentrations (50 and 100 μM) as shown in **Supplementary Figure 3**. The samples were collected at various time points: 0, 2, 4, 8, 12, and 24 h after Cd stress, and used for total RNA extraction and subsequent analysis. For the generation of transformed Chinese cabbage plants, the floral dip method and vernalization infiltration technique were employed (Chen et al., 2021).

### 2.2 Gene clone and plasmid construction

The CDS was cloned into the pRS416-GFP vector to construct the overexpression vectors. NSR1 and VLD1 genes were cloned into the pRS423 vector in fusion with the RFP sequence. BrMYB116 was cloned from the cDNA of Chinese cabbage, while the coding sequences of ScFIT3, NSR1, and VLD1 were amplified using the cDNA of yeast (JRY472) with specific primers. Subsequently, the amplified sequences were inserted into pRS416-GFP and pRS423 in fusion with the RFP sequence. For expression constructs of BrMYB116 and ScFIT3, DNA was PCR amplified from genomic DNA of Chinese cabbage (Cv. Guangdongzao) and yeast (JRY472), respectively, with specific primers. The amplified sequences were subsequently inserted into the binary vector pCambia3300 to yield pBrMYB116::BrMYB116 and pScFIT3::ZmO2L1.

### 2.3 Total RNA extraction, RT-qPCR, and quantitative real-time PCR analysis

Total RNA was extracted from Chinese cabbage tissues using TRIzol. For yeast RNA extraction, the M5-EASTspin Yeast RNA Rapid Kit was employed, as previously described. The RNA was then used for cDNA synthesis. For qRT-PCR analysis, the SYBER premix-Ex-Taq kit was used. The experiment was performed using three biological replicates.

### 2.4 Tolerance assay and growth curve

Yeast cells transformed with either the BrMYB116 and ScFIT3-expressing pRS416-GFP vectors, along with those transformed with the empty pRS416-GFP (WT) vector, were cultured overnight in an SC (URA^−^) fluid medium at 30°C. The cell solutions were then diluted to achieve an OD_600_ of approximately 0.1 and allowed to grow until reaching an OD_600_ of 0.3. After precisely adjusting the OD_600_ to 0.3, the cell solutions were fivefold diluted and spotted onto plates with or without 75 μM of Cd. The cells were incubated at 30°C for 2 days. The experiments were repeated three times. The growth curve of BrMYB116 and WT was conducted after cultivation in a liquid SC (URA^−^) medium at 30°C. The solutions were diluted to an OD_600_ of approximately 0.1 and then further incubated to reach an OD_600_ of approximately 0.3. After precisely adjusting the OD_600_ to 0.3, the solution was treated with 75 μM of Cd for 12 h. Subsequently, OD_600_ was measured every 2 h to monitor the growth pattern.

### 2.5 Subcellular localization assay

The yeast cells co-transformed with pRS416-GFP-expressing vectors or BrMYB116 ScFIT3- and pRS423-expressing vectors NSR1 and VLD1 in fusion with RFP were cultured overnight in an SC (URA^−^ and HIS^−^) fluid medium at 30°C. The yeast cells transformed with an empty pRS416-GFP vector were also cultured overnight in an SC (URA^−^) fluid medium at 30°C. Then, the solutions were diluted to an OD_600_ of approximately 0.1 and allowed to grow until reaching an OD_600_ of approximately 0.3. After adjusting the OD_600_ to exactly 0.3, the solutions were treated with or without 75 μM of Cd. After incubating for about 12 h at 30°C, the yeast cells were observed under a Zeiss 300 confocal microscope.

### 2.6 RNA-seq library construction and sequencing

The yeast cells expressing *BrMYB116* were treated with 75 μM Cd for 14 h, and then the cells were harvested and sent to Noveogen for transcriptome analysis. Three independent biological replicates were performed. Briefly, RNA integrity was tested using the RNA Nano 6000 Assay Kit on a Bioanalyzer 2100 system (Agilent Technologies, CA, USA). Total RNA was used as the input material for RNA sample preparation. mRNA was purified using poly-T oligo-attached magnetic beads. Fragmentation was performed using divalent cations under elevated temperatures in a first-strand synthesis reaction buffer. The first-strand cDNA was synthesized using a random hexamer primer and M-MuLV reverse transcriptase (RNase H). Subsequently, second-strand cDNA synthesis was carried out using DNA polymerase I and RNase H. Remaining overhangs were converted to blunt ends by exonuclease/polymerase. After the adenylation of the 3′ DNA fragments, adaptors with hairpin loop structures were prepared for hybridization. To select cDNA fragments within the preferred length range of 370–420 bp, the library fragments were purified with an AMPure XP system (Beckman Coulter, Beverly, USA). Subsequently, PCR was carried out with Phusion high-fidelity DNA polymerase, universal PCR primers, and the Index (X) Primer. The resulting PCR products were purified using the AMPure XP system, and library quality was evaluated using an Agilent Bioanalyzer 2100 system. The clustering of index-coded samples was carried out on a cBot Cluster Generation System using a TruSeq PE Cluster Kit v3-cBot-HS (Illumina). After cluster generation, the library was sequenced on an Illumina Novaseq platform, and 150-bp paired-end reads were generated.

### 2.7 Promoter activity assay

The promoter activity assay was carried out in tobacco as previously described (He et al., 2016). Briefly, all reporter vectors were transformed into GV3101 strains. A single clone was inoculated into YEP medium with rifampicin and kanamycin and grown at 28°C overnight. The cells were collected and resuspended, and the OD_600_ was adjusted to 1. The cells were incubated at room temperature for about 2 h, after which, they were infiltrated into 4-week-old tobacco leaves. Subsequently, these leaves were placed in a dark growth chamber overnight, followed by exposure to light for 2 days, as previously described. The Dual-Luciferase Reported Assay system (catalog No. E1910; Promega) was used for the quantification of luciferase values.

### 2.8 ChIP-qPCR assay

ChIP in yeast was performed according to an established method with minor modifications. Briefly, 100 ml of yeast cells (with an OD of 1.0) were cross-linked by 1% of formaldehyde for 20 min at 30°C. The sonicated protein–DNA complex was immunoprecipitated with or without (negative control) anti-flag antibody. The DNA from these complexes was then used for ChIP-qPCR assay. ChIP signals were calculated as previously described. All primers used for qPCR are listed in **Supplementary Table S1**.

### 2.9 Cd accumulation measurement

The content of Cd in Chinese cabbage seedlings was measured using an ICP-MC instrument as previously described (Gong et al., 2003).

## 3 Result

### 3.1 Verification of Cd tolerance by BrMYB116 in the yeast system

**Figure 1:**
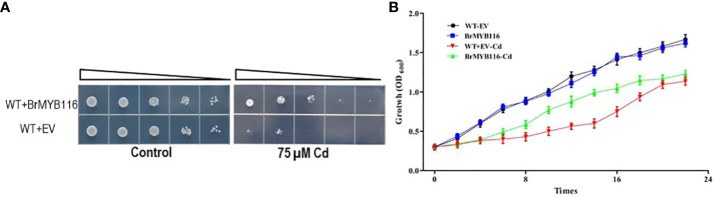
Assessment of Cd tolerance in yeast cells. **(A)** Yeast dilution assay comparing the wild-type and BrMYB116-expressing strains in SD medium. Triangles represent serial 10-fold dilutions (starting concentration of 0.3 OD_600_). **(B)** Both the wild-type yeast strain and transgenic cells were grown at 30°C in SD liquid media and subjected to 75 µM of CdCl_2_ at an OD_600_ of 0.3. Cell density was monitored with the absorbance at 600 nm at 12, 14, 16, 18, 20, and 22 h after the Cd treatment. Error bars indicate ± SD from three independent experiments. Statistical significance was assessed using the Student’s t-test. **p < 0.01; ***p < 0.001.

In our previous report (He et al., 2022), a number of Cd response genes (CRGs) from the Chinese cabbage cultivar “Guangdongzao” have been identified via transcriptome profile analyses. As yeast is a common eukaryotic model for studying Cd-resistant mechanisms, it was employed in this study to investigate the CRGs of Chinese cabbage involved in Cd tolerance (He et al., 2022). The genes were fused with a glycerol phosphate dehydrogenase (GPD) promoter in the yeast expressing recombination vector pRS416 to yield pGPD::CRGs, which were then transformed into budding yeast (cv JRY472). Serial dilution assays were conducted to evaluate Cd tolerance. The results show that the strains expressing *BrMYB116* demonstrated enhanced growth compared with the wild-type JRY472 with an empty vector (**Figure 1A**). Growth curves in yeast expressing *BrMYB116*, with or without Cd stress, revealed that overexpressing cells displayed a Cd tolerance phenotype, particularly between 8 and 16 h post-Cd treatment (**Figure 1B**).

### 3.2 BrMYB116 specifically accumulates in the nucleus under Cd stress

**Figure 2:**
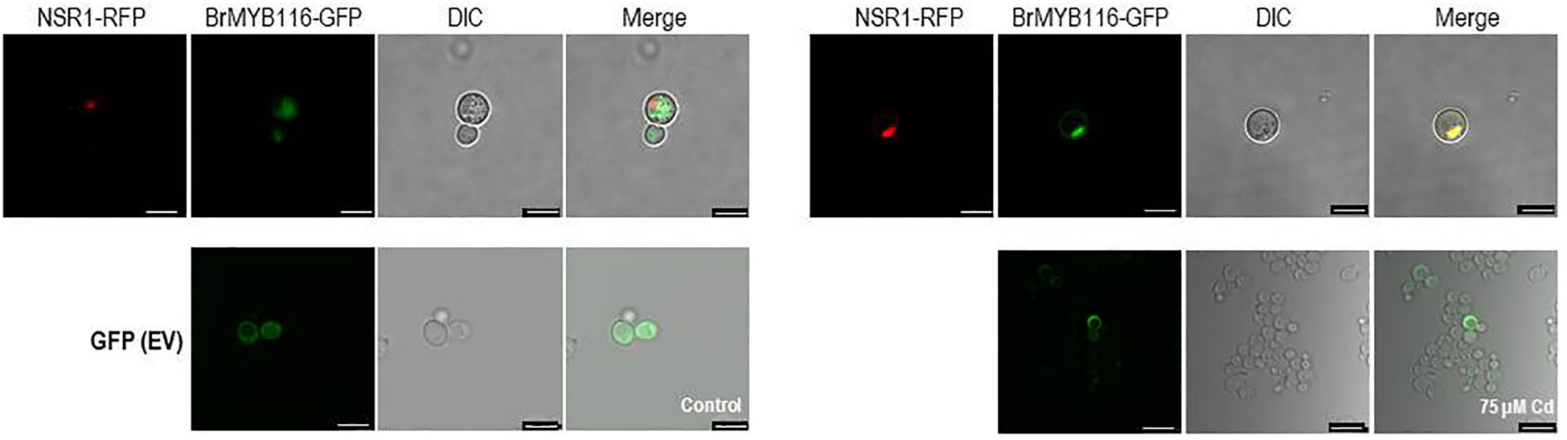
Subcellular localization of BrMYB116 tagged with GFP and expressed in yeast cells with or without 75 µM of CdCl_2_. The nuclear-localized NSR1 gene was used as the positive control. The images were captured using RFP, GFP, and DIC channels or as merged images of two channels. Scale bar: 10 μm.

To explore the specific role of BrMYB116 in Cd stress tolerance, the CDS was fused with GFP and then expressed in yeast cells. The pRS423 vector, expressing the nuclear-localized yeast gene NSR1 in fusion with RFP, was used as a positive control (Yan and Melese, 1993), while EV (empty vector; pRS416-EGFP) was used as a negative control. In the absence of Cd, BrMYB116 was predominately localized in the cytoplasm. However, when exposed to 75 µM of CdCl_2_, BrMYB116 was translocated into the nucleus, while the GFP signal of empty vectors remained unaffected by Cd stress (**Figure 2**), indicating the specific nuclear localization of BrMYB116 under Cd stress. In contrast, when BrMYB116 fused with GFP was expressed in *Arabidopsis* protoplasts, no noticeable difference in cellular localization was observed regardless of Cd presence or absence (**Supplementary Figure 1**). While BrMYB116 is consistently localized in the nucleus of *Arabidopsis* protoplasts irrespective of external Cd levels (**Figure 1**), its specific nuclear localization in yeast suggests a potential regulatory role in Cd stress tolerance at the molecular level. These results also imply that BrMYB116 may interact with the *cis*-acting elements of its target genes in the nucleus, which are essential for Cd stress response in yeast.

### 3.3 Differentially expressed gene (DEGs) and RNA-seq validation

**Figure 3:**
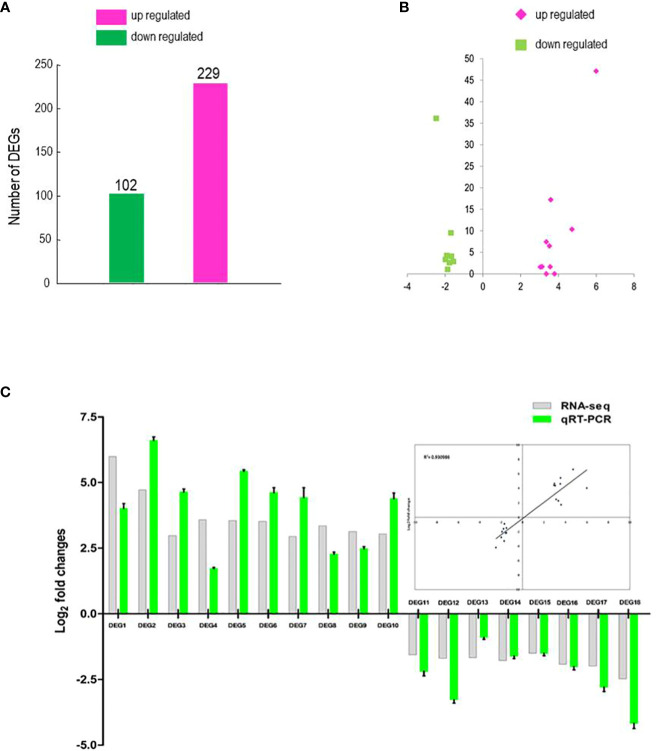
RNA-seq analysis and DEG identification. **(A)** Comparison of DEG numbers in BrMYB116-expressing cells versus wild-type (WT) cells. **(B)** Volcano plot of the selected DEGs (log2 values >3 or <−1.5) from **(A)**. The x-axis represents the fold change shown as log2 values; The y-axis represents the adjusted p-value shown as −log10. Magenta represents increased expression; green represents decreased expression relative to WT. **(C)** qPCR validation of the selected DEGs (relative to ACT2) in the same batch of the Cd-treated yeast cells from the RNA-seq data. Error bars indicate ± SD from three independent experiments.

The response of BrMYB116 to Cd stress may be due to changes in transcript abundance of genes regulated by BrMYB116. An RNA-seq analysis was carried out on Cd-treated yeast cells transformed with BrMYB116-expressing vectors to examine whether the gene expression pattern has changed. A total of 331 DEGs with statistically significant changes [log2 (FoldChange) ≤1 or ≥1 and adjusted p-value ≤0.05] were selected (**Supplementary Table S2**; **Figure 3A**). GO enrichment analysis showed that numerous DEGs are involved in transporter and transmembrane transporter activity (**Supplementary Table S3**) indicating that BrMYB116 might enhance Cd tolerance by regulating Cd ion transport. Given that BrMYB116 could regulate more than one target gene to confer Cd tolerance, we focused on a smaller group of DEGs with more pronounced expression changes [log2(FoldChange) ≤−1.5 or ≥3). These DEGs, labeled as DEG1 to DEG18 in descending order of expression in the clustering analysis map, include gene-CENPK1137D_4855, gene-CENPK1137D_521, gene-CENPK1137D_574, gene-CENPK1137D_5283, gene-CENPK1137D_2397, gene-CENPK1137D_417, gene-CENPK1137D_2396, gene-CENPK1137D_5368, gene-CENPK1137D_699, gene-CENPK1137D_138, gene-CENPK1137D_3368, gene-CENPK1137D_3854, gene-CENPK1137D_4708, gene-CENPK1137D_3277, gene-CENPK1137D_4991, gene-CENPK1137D_2539, gene-CENPK1137D_4598, and gene-CENPK1137D_3634 (**Figure 3B**).

### 3.4 Overexpression of DEG5 enhances Cd tolerance in yeast

**Figure 4:**
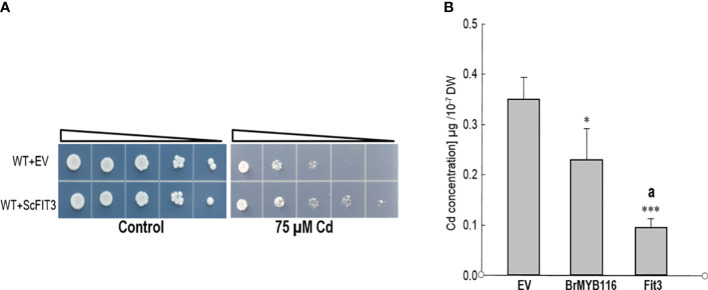
Assessment of Cd tolerance and accumulation. **(A)** Dilution bioassay comparing the wild-type yeast strain and the strain overexpressing *ScFIT3*. Triangles represent serial 10-fold dilutions (starting OD_600_ of 0.3). The depicted assay is representative of three consistent experiments. **(B)** Cd concentrations in yeast cells. Yeast strains were grown on SC solid plates with or without 75 µM of CdCl_2_ at 30°C for 2 days. Cells were collected in liquid SC with 75 µM of Cd, and OD_600_ was recorded before atomic absorption spectrometer measurements. Error bars indicate the ± SD of three independent experiments. The significance of the difference was evaluated using Student’s t-test between the BrMYB116-expressing strains and the strains with the empty vector (*p < 0.05; ***p < 0.001) and between the BrMYB116-expressing strains and the ScFIT3-expressing strains (^a^p < 0.05).

To validate transcriptome analysis, 18 DEGs were selected and used for quantitative real-time PCR as presented in **Figure 3C**. The qRT-PCR results were consistent with the RNA-seq data for most genes (**Figure 3C**). To investigate their functions, these 18 DEGs were expressed in yeast cells under the control of a GPD promoter. Under Cd stress (75 µM), only yeast cells overexpressing DEG5 (CENPK1137D_2397), which encodes a putative member of the facilitator of iron transport 3 (FIT3), exhibited more substantial growth than the wild-type control (**Figure 4A**; **Supplementary Figure 2**).

This finding indicates that the overexpression of DEG5 alone is sufficient to confer Cd tolerance, underscoring its vital role in managing Cd stress. Furthermore, assessments of Cd content in yeast cells revealed reductions in Cd accumulation for both BrMYB116 and ScFIT3 compared to JRY472 (EV(WT)) (**Figure 4B**) indicating that the Cd tolerance phenotype mediated by BrMYB116 and ScFIT3 is mainly attributed to reductions in Cd accumulation. Notably, JRY472 (ScFIT3) showed greater reductions in Cd levels than JRY472 (BrMYB116) (**Figure 4B**). Given that diminished Cd accumulation is a key indicator of Cd tolerance, these findings suggest ScFIT3 as the potential primary target gene regulated by the transcription factor BrMYB116 in the Cd tolerance pathway.

### 3.5 ScFIT3 is ubiquitously present in yeast cell vacuoles under Cd stress

**Figure 5:**
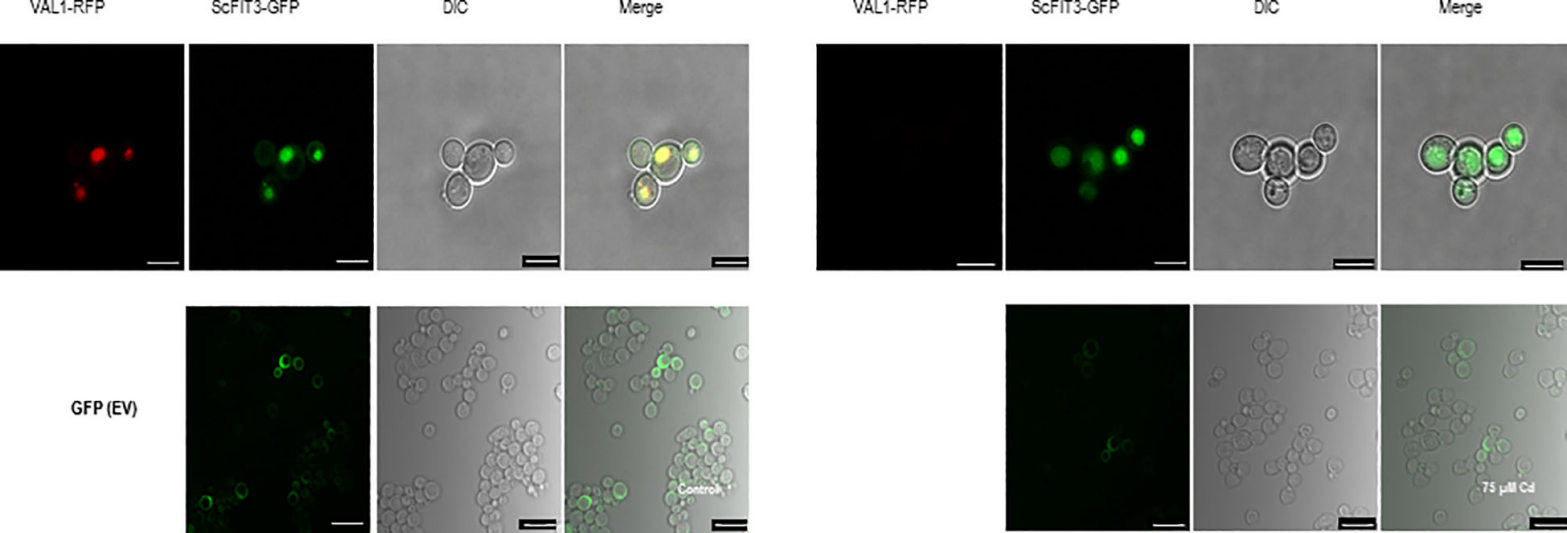
Subcellular localization of ScFIT3 tagged with GFP and expressed in yeast cells with or without 75 µM of CdCl_2_. Vacuole-localized gene VLD1 was used as a positive control. The images were captured from the RFP, GFP, and DIC channels or as merged images of two channels. Scale bar: 10 μm.

ScFIT3, a member of the facilitator of iron transporter family, is known to transfer Cd ions in addition to iron ions. We investigated the subcellular localization of ScFIT3 by fusing its CDS with GFP and expressing it in yeast cells. The pRS423-vector-expressing vacuole-localized gene VLD1 was fused with RFP as the positive control, and pRS416-GFP was used as the negative control (Huh et al., 2003). Under Cd stress, ScFIT3 was localized in the vacuole. However, ScFIT3 was distributed throughout the cell upon Cd treatment (**Figure 5**). The absence of VLD1 fused with RFP in yeast cells under Cd stress suggests that Cd might have disrupted its expression. ScFIT3 changed its subcellular localization when treated with Cd stress. Under normal conditions without Cd stress, ScFIT3 may play a role in facilitating the transport of iron ions in the vacuole. However, in the presence of Cd, ScFIT3 appears to facilitate the transport of the Cd ions from the vacuole and throughout the whole cell. Cd content measurements (**Figure 4B**) showed that the altered localization of ScFIT3 might contribute to the exclusion of Cd ions from the cell membrane.

### 3.6 BrMYB116 is associated with the promoter of ScFIT3

**Figure 6:**
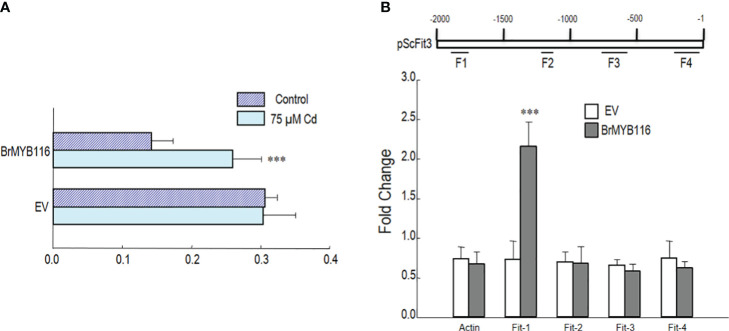
BrMYB116 activate and bind the fragment of ScFIT3 promoter under Cd stress. **(A)** BrMYB116 activation of ScFIT3 promoter (including 5′ UTR) determined by infiltration mediated transient expression assay. X axis is the ratio of LUC to rLUC activity two days after infiltration. Numbers indicate position of starting nucleotide of each BOXS2 relative to translation start. Error bars indicate ± SD of three biological repeats. P value of student’s t test: BrMYB116 compared with empty vector. ***P<0.001. **(B)** BrMYB116 bind the promoter of ScFit3. ChIP-qPCR was performed to test in vivo interaction of promoters (including 5’UTR) with BrMYB116 in cells from WT, WT(*BrMYB116-GFP*) treated with 75 μM Cd. DNA fragments from different parts of promoters were tested (labeled with F1-F4). CP (crossing point) value of immuno-precipitated DNA fractions with GFP or no antibody control (NoAb) normalized to CP value of input DNA fractions for the same qPCR assay. Y axis is the ChIP signals calculated as the enrichment relative to the no-antibody control (No Ab). Error bars indicate ± SD from three independent experiments.

To explore the interaction between BrMYB116 and ScFIT3, a promoter–luciferase fusion was created by combining the 2-kb promoter of the firefly luciferase gene, including the 5′ UTR fragment from ScFIT3, into a promoter–*luc* fusion construct. Both *p35S::BrMYB116* and empty vector (control) constructs were transiently introduced into tobacco leaves. In transactivation assay, enhanced expression of pScFIT3-*luc* via *p35S::BrMYB* was observed under Cd treatment compared to that without Cd (**Figure 6A**). These results indicated that BrMYB116 specifically activated pScFIT3 under Cd stress. A ChIP-qPCR experiment was carried out to confirm the interaction of BrMYB116 with the promoter fragments in transgenic yeast cells producing GFP-tagged BrMYB116. Following the immunoprecipitation with an anti-GFP antibody, four primer pairs (F1, F2, F3, and F3) were used for targeting different sections (500 bp) of the corresponding promoter (**Figure 6B**). The findings indicated a positive interaction for BrMYB116 at F1 (**Figure 6B**), but not with other fragments, including ACT1 (XM_009117825, NCBI) used as a negative control. This suggests that BrMYB116 binds specifically to a certain region of the ScFIT3 promoter to regulate its expression.

### 3.7 BrMYB116 is induced in Chinese cabbage by Cd stress

**Figure 7:**
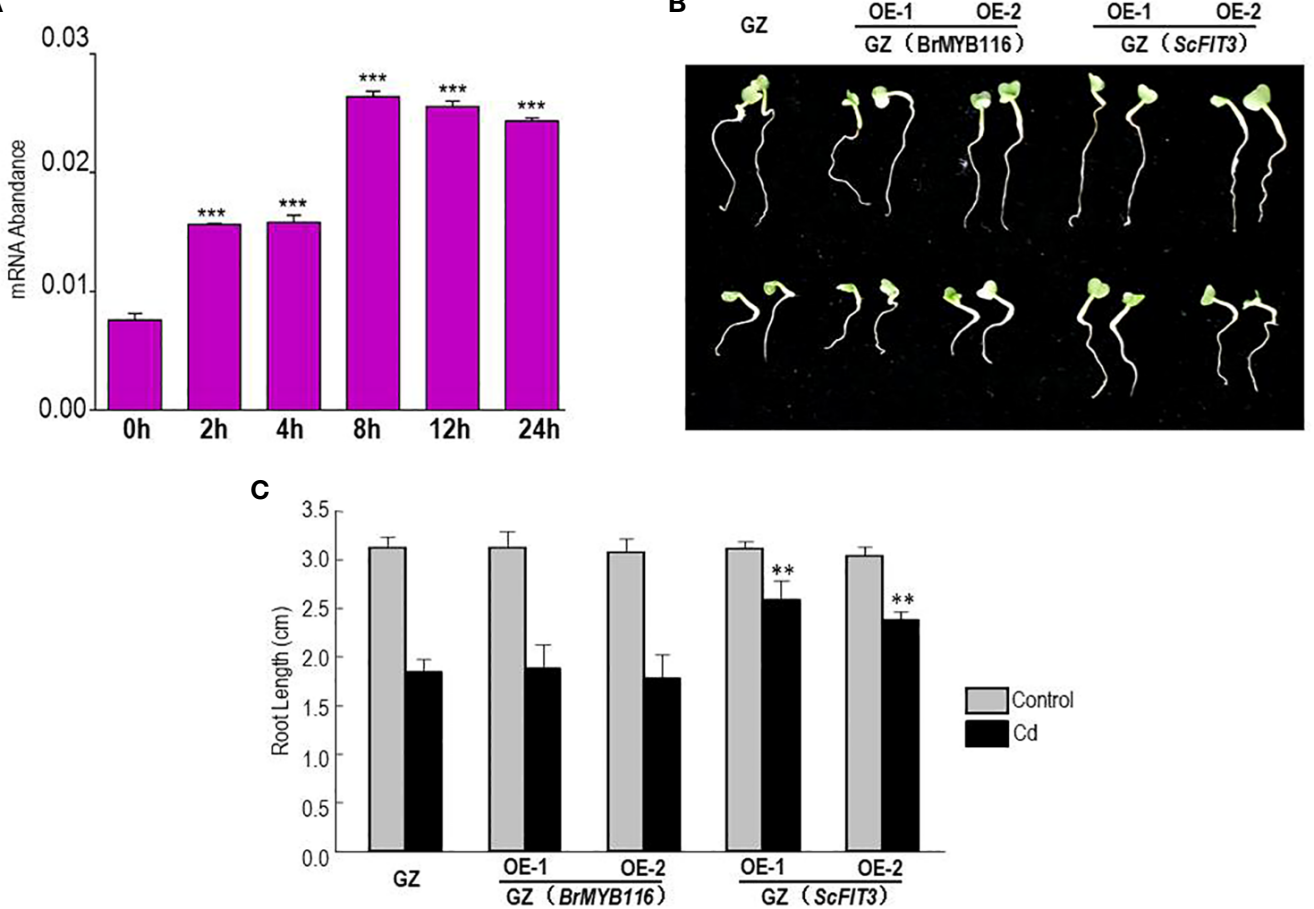
*BrMYB116* mRNA abundance and Cd tolerance assessment in Chinese cabbage. **(A)** *BrMYB116* transcript abundance in Chinese cabbage seedlings (relative to *BrACT2* control) determined by RT-qPCR. Six-day-old seedlings were exposed to 75 µM of CdCl_2_. Error bars indicate ± SD from three independent experiments. The significance of the difference was evaluated using Student’s t-test. ***p < 0.001. **(B)** Chinese cabbage plants germinated for 2 days were transferred to hydroponic MS media without or with 50 µM of CdCl_2_ for another 3 days. The displayed results are representatives of three reproducible experiments. OE-1 and OE-2 are two independent transgenic lines. **(C)** Average root length of seedlings cultured under the same growth condition as shown in **(B)** The root length of three seedlings of each class was measured as the mean value. The significance of the difference was evaluated using Student’s t-test between the BrMYB116 and ScFIT3-expressing plants and the wild-type control (**p < 0.01; ***p < 0.001).

To further explore the role of BrMYB116 in Cd stress tolerance in Chinese cabbage, the expression of *BrMYB116* was tested in response to Cd stress. Chinese cabbage seedlings were grown in MS nutrient solution with or without 200 µM of CdCl_2_, and then the samples were collected at different time intervals. The qRT-PCR results revealed a steady increase in BrMYB116 mRNA levels, with the highest expression observed at the eighth hour post-Cd treatment (**Figure 7A**). These findings indicate that the expression of BrMYB116 was activated by Cd stress suggesting its involvement in Cd responses within the Chinese cabbage. Subsequently, we transferred BrMYB116 into the “Guangdongzao” cultivar and generated transgenic lines. After confirming the successful overexpression of BrMYB116 via RT-qPCR, two independent lines were selected for further analyses of the Cd tolerance phenotype. However, these lines did not exhibit enhanced Cd tolerance in comparison to wild-type plants, as depicted in **Figures 7B, C**. As a transcription factor activated by Cd stress, the downstream target gene in the Cd tolerance pathway might be missing in Chinese cabbage.

### 3.8 FIT3 reduces Cd accumulation in Chinese cabbage

**Figure 8:**
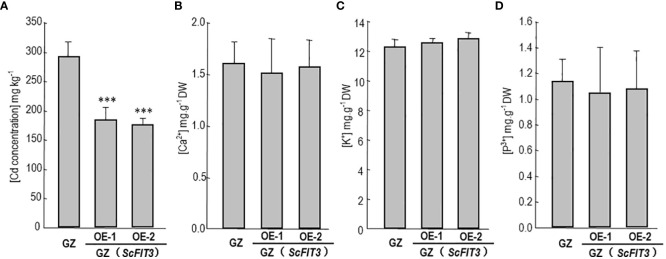
Cd content and some nutrition ion contents in the Chinese cabbage. The content of Cd **(A)**, Ca^2+^ **(B)**, K^+^ **(C)**, p^3+^ **(D)** in the wild-type and ScFIT3 transgenic Chinese cabbage. Error bars indicate ± SD of three biological repeats. P value of student’s t test: BrMYB116 or ScFIT3 transgenic plants compared with the wild-type control. ***P<0.001.

To explore the function of FIT3 in preventing Cd accumulation in plants, we transferred ScFIT3 to the “Guangdongzao” cultivar of Chinese cabbage. After confirming the successful integration of ScFIT3 via RT-PCR (**Supplementary Figure 4**), two independent lines were selected to explore its involvement in Cd stress tolerance and uptake. Under Cd stress, transgenic seedlings exhibited improved growth and longer root lengths compared to the wild-type control (**Figures 7B, C**). Moreover, the Cd content was significantly reduced in these two transgenic lines compared to the wild-type control (**Figure 8A**) indicating that the improved growth in the transgenic lines was attributed to reduced Cd accumulation and toxicity. Recognizing the importance of Chinese cabbage as a dietary vegetable, we also analyzed the content of several essential nutritional ions in transgenic seedlings. As shown in **Figures 8B–D**, the contents of K, Cd, and P ions were indistinguishable between the transgenic seedlings and the wild-type control suggesting that the expression of FIT3 did not affect the uptake of the primary nutritional ions.

## 4 Discussion

Transcription factors are pivotal molecular entities regulating the expression of downstream genes in organisms, which can translocate into the cell nucleus in response to specific regulatory needs. Thus, before conducting RNA-seq analysis in yeast, we investigated the subcellular localization pattern of BrMYB116 under the treatment of Cd. The nuclear localization of BrMYB116 under Cd exposure prompted us to identify potential downstream genes regulated by BrMYB116, ultimately contributing to enhanced Cd tolerance in yeast. Without knowing the subcellular localization of BrMYB116, the gene expression profile analysis would have been less reliable. Consequently, the RNA-seq was conducted, which led to the identification of FIT3.

FIT3 is a facilitator of iron transport primarily localized in the vacuole in the absence of Cd. However, upon exposure to Cd, the vacuole localization of ScFIT3 disappeared. It is hypothesized that under Cd stress conditions, ScFIT3 pivots its function toward the transport of Cd ions rather than its typical role in facilitating iron transport. This shift in ion specificity could lead to ScFIT3 vacating the vacuole consequently reducing Cd content within the yeast cell.

As a unicellular organism, yeast serves as a valuable model for understanding various biological processes. Given the demonstrated capacity of FIT3 to reduce Cd content within yeast cells, we sought to investigate its impact on Cd accumulation in Chinese cabbage. To this end, we introduced ScFIT3 into Chinese cabbage and examined its effects. The result revealed that ScFIT3 also led to a notable reduction in Cd content in Chinese cabbage highlighting an essential role in addressing Cd contamination especially in leafy vegetables. Notably, the lack of a homologous FIT3 gene in Chinese cabbage, along with the association of BrMYB116 with the promoter region of FIT3, emphasizes the potential regulatory role of BrMYB116 in Cd tolerance pathways. Given the ability of FIT3 to mitigate Cd toxicity in Chinese cabbage and its apparent regulation by the transcription factor BrMYB116, it is plausible that FIT3 represents a real target candidate gene within the Cd tolerance pathway, and its loss in the evolutionary process may explain the absence of a functional homolog in Chinese cabbage. Many transcription factors are identified as pivotal in Cd tolerance using a yeast system. However, it is noteworthy that many of these transcription factors, when assessed in their native species, fail to manifest the same Cd tolerance phenotype. For example, AtOXS2 induces Cd stress tolerance in yeast but does not enhance Cd tolerance in *Arabidopsis* (Blanvillain et al., 2011).

To delve deeper into this phenomenon, we tested the homologous genes of AtOXS2 from maize, including ZmOXS2b and ZmO2L, in response to Cd stress. Intriguingly, both ZmOXS2b and ZmO2L displayed a remarkable ability to confer Cd tolerance in *Arabidopsis* (He et al., 2016). Furthermore, we found that both ZmOXS2b and ZmO2L can bind and activate the promoter of CIMT1, a phenomenon not observed with AtOXS2 (He et al., 2016). These findings suggest a substantial divergence among the family members of these transcription factors during the evolutionary processes.

In the pursuit of comprehending the mechanisms underlying Cd tolerance pathways in plants and microorganisms, many transcription factors and family genes are involved in Cd stress tolerance. However, the molecular mechanisms remain elusive for a significant portion of these transcription factors, as their downstream target genes have not been completely identified. Following the identification of BrMYB116 as a contributor to Cd tolerance in yeast, we embarked on translational research endeavors by introducing the BrMYB116 gene into Chinese cabbage. Although it was evident that the expression of BrMYB116 responded to the Cd treatment (**Figure 7A**), no discernible disparity of Cd tolerance was observed between the BrMYB116-overexpressing line and the wild-type control (**Figures 7B, C**). Consequently, further investigation of its target genes in Chinese cabbage was deemed unnecessary. Instead, our focus shifted toward unraveling the Cd tolerance mechanism of BrMYB116 in yeast, which led to the discovery of ScFIT3. Our results showed that ScFIT3 enhanced Cd resistance and substantially reduced Cd accumulation within yeast cells.

Prior to introducing ScFIT3 into Chinese cabbage, we initially sought to elucidate the function of FIT3 in Chinese cabbage by overexpressing its homologous gene, referred to as “BrFIT3.” However, a comprehensive examination of the protein sequence of ScFIT3 through a sequence similarity search in the National Center for Biotechnology Information (NCBI) database revealed the absence of any homologous protein or gene counterpart in Chinese cabbage or other plants. Consequently, we resorted to generating transgenic Chinese cabbage lines expressing ScFIT3. Although BrMYB116 did not confer Cd resistance in Chinese cabbage, our investigations into FIT3, initially conducted in yeast, yielded noteworthy outcomes. It was evident that ScFIT3 could enhance Cd resistance and decrease Cd accumulation in Chinese cabbage. This progression of research brings our focus back to Chinese cabbage. Through a cyclic transformation process involving Chinese cabbage to yeast, and yeast to Chinese cabbage, we have successfully established the Cd tolerance pathway orchestrated by MYB116, with FIT3 serving as a pivotal player in this intricate regulatory network.

## 5 Conclusions

**Figure 9:**
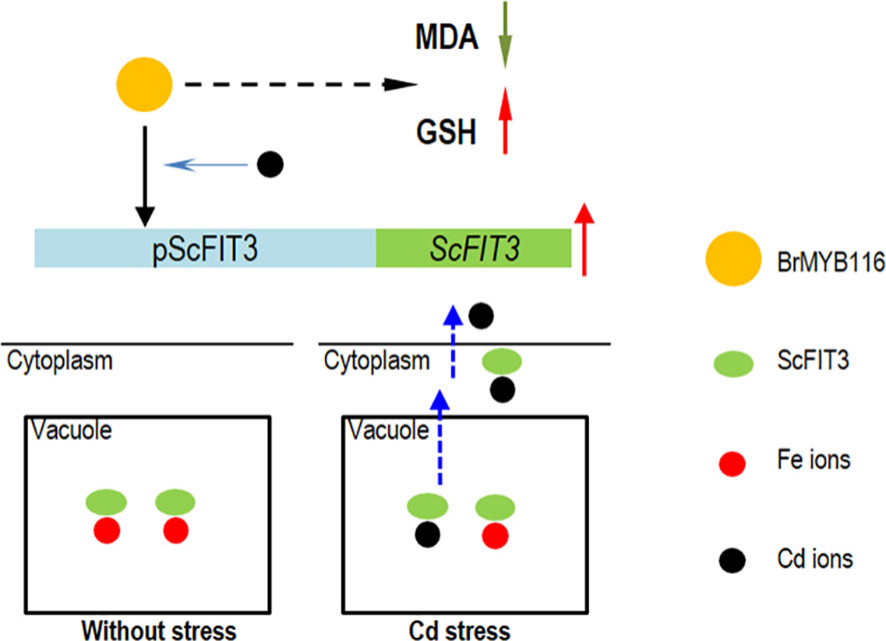
The hypothesis model of BrMYB116 regulating Cd tolerance in yeast cells. The yellow circle indicates BrMYB116, ScFIT3 is shown as green ovals, the Fe ions are indicated as red circles, and the Cd ions are indicated as black circles.

Cd toxicity poses a threat to human health via food contamination. Therefore, compared with other methods for the remediation of Cd contamination, a novel Cd transporter that can exclude Cd ions from the cells or the plants is imperative and can be easily used for the genetic engineering of vegetables and crops. In this study, we identified a classical MYB transcriptional factor from Chinese cabbage, BrMYB116, which is associated with the iron ion transporter gene, *ScFIT3*, of yeast and facilitates the exclusion of Cd in both yeast and Chinese cabbage. After a protein sequence blast in NCBI and Phytozome, the homologous genes of FIT3 were not found in other plant species; it is possible that FIT3 was lost during the evolutionary process. The ancient heavy metal resistance mechanism regulated by FIT3 and MYB116 might be elucidated and utilized by plants to remediate Cd toxicity and exclude Cd from plant tissues (**Figure 9**).

## Data availability statement

The data presented in the study are deposited in the NCBI repository, accession number SAMN31875212.

## Author contributions

AA: Data curation, Formal analysis, Methodology, Software, Supervision, Writing – original draft, Writing – review & editing. CY: Investigation, Software, Writing – original draft. BC: Conceptualization, Data curation, Methodology, Writing – original draft, Writing – review & editing. LW: Conceptualization, Investigation, Methodology, Writing – original draft, Writing – review & editing. LH: Funding acquisition, Software, Supervision, Validation, Visualization, Writing – original draft, Writing – review & editing. JG: Conceptualization, Investigation, Resources, Software, Visualization, Writing – original draft, Writing – review & editing.

## References

[B1] Agarwal P. Mitra M. Banerjee S. Roy S. (2020). MYB4 transcription factor, a member of R2R3-subfamily of MYB domain protein, regulates cadmium tolerance via enhanced protection against oxidative damage and increases expression of PCS1 and MT1C in Arabidopsis. Plant Sci. 297, 110501. doi: 10.1016/j.plantsci.2020.110501 32563471

[B2] Baldoni E. Genga A. Cominelli E. (2015). Plant MYB transcription factors: their role in drought response mechanisms. Int. J. Mol. Sci. 16, 15811–15851. doi: 10.3390/ijms160715811 26184177 PMC4519927

[B3] Bashir S. Hussain Q. Shaaban M. Hu H. Q. (2018). Efficiency and surface characterization of different plant derived biochar for Cadmium (Cd) mobility, bioaccessibility and bioavailability to Chinese cabbage in highly contaminated soil. Chemosphere 211, 632–639. doi: 10.1016/j.chemosphere.2018.07.168 30098558

[B4] Bertin G. Averbeck D. (2006). Cadmium: cellular effects, modifications of biomolecules, modulation of DNA repair and genotoxic consequences (a review). Biochimie 88, 1549–1559. doi: 10.1016/j.biochi.2006.10.001 17070979

[B5] Blanvillain R. Wei S. Wei P. Kim J. H. Ow D. W. (2011). Stress tolerance to stress escape in plants: role of the OXS2 zinc-finger transcription factor family. EMBO J. 30, 3812–3822. doi: 10.1038/emboj.2011.270 21829164 PMC3173794

[B6] Cai S. Y. Zhang Y. Xu Y. P. Qi Z. Y. Li M. Q. Ahammed G. J.. (2017). : HsfA1a upregulates melatonin biosynthesis to confer cadmium tolerance in tomato plants. J. Pineal Res. 62 (23). doi: 10.1111/jpi.12387 28095626

[B7] Chen D. He L. Lin M. Jing Y. Liang C. Liu H.. (2021). A ras-related small GTP-binding protein, RabE1c, regulates stomatal movements and drought stress responses by mediating the interaction with ABA receptors. Plant Sci. 306, 110858. doi: 10.1016/j.plantsci.2021.110858 33775364

[B8] Chen X. Wu Y. Yu Z. Gao Z. Ding Q. Shah S. H. A.. (2023). BcMYB111 responds to bcCBF2 and induces flavonol biosynthesis to enhance tolerance under cold stress in non-heading Chinese cabbage. Int. J. Mol. Sci. 24, 8670. doi: 10.3390/ijms24108670 37240015 PMC10217907

[B9] Chen H. C. Zhang S. L. Wu K. J. Li R. He X. R. He D. N.. (2020). The effects of exogenous organic acids on the growth, photosynthesis and cellular ultrastructure of Salix variegata Franch. Under Cd stress. Ecotoxicology Environ. Saf. 187, 109790. doi: 10.1016/j.ecoenv.2019.109790 31639642

[B10] Cui W. N. Wang H. T. Song J. Cao X. Rogers H. J. Francis D.. (2017). et al: Cell cycle arrest mediated by Cd-induced DNA damage in Arabidopsis root tips. Ecotoxicology Environ. Saf. 145, 569–574. doi: 10.1016/j.ecoenv.2017.07.074 28800532

[B11] Dong J. Cao L. Zhang X. Y. Zhang W. H. Yang T. Zhang J. Z.. (2021). An R2R3-MYB transcription factor rmMYB108 responds to chilling stress of Rosa multiflora and conferred cold tolerance of Arabidopsis. Front. Plant Sci. 12. doi: 10.3389/fpls.2021.696919 PMC8353178 34386027

[B12] Dubos C. Stracke R. Grotewold E. Weisshaar B. Martin C. Lepiniec L. (2010). MYB transcription factors in Arabidopsis. Trends Plant Sci. 15, 573–581. doi: 10.1016/j.tplants.2010.06.005 20674465

[B13] Gallego S. M. Pena L. B. Barcia R. A. Azpilicueta C. E. Lannone M. F. Rosales E. P.. (2012). Unravelling cadmium toxicity and tolerance in plants: Insight into regulatory mechanisms. Environ. Exp. Bot. 83, 33–46. doi: 10.1016/j.envexpbot.2012.04.006

[B14] Gao S. Zhang Y. L. Yang L. Song J. B. Yang Z. M. (2014). AtMYB20 is negatively involved in plant adaptive response to drought stress. Plant Soil 376, 433–443. doi: 10.1007/s11104-013-1992-6

[B15] Gong J. M. Lee D. A. Schroeder J. I. (2003). Long-distance root-to-shoot transport of phytochelatins and Cadmium in *Arabidopsis* . Proc. Natl. Acad. Sci. 100, 10118–10123. doi: 10.1073/pnas.1734072100 12909714 PMC187785

[B16] He L. Ma X. Li Z. Jiao Z. Li Y. Ow D. W. (2016). Maize OXIDATIVE STRESS2 homologs enhance cadmium tolerance in Arabidopsis through activation of a putative SAM-dependent methyltransferase gene. Plant Physiol. 171, 1675–1685. doi: 10.1104/pp.16.00220 27208260 PMC4936553

[B17] He L. Yuan C. Li X. Li C. Li Y. Chen D.. (2022). Metabolomics analysis reveals different mechanisms of cadmium response and functions of reduced glutathione in cadmium detoxification in the Chinese cabbage. Plant Growth Regul. 98, 289–305. doi: 10.1007/s10725-022-00860-7

[B18] Hu S. B. Yu Y. Chen Q. H. Mu G. M. Shen Z. G. Zheng L. Q. (2017). OsMYB45 plays an important role in rice resistance to cadmium stress. Plant Sci. 264, 1–8. doi: 10.1016/j.plantsci.2017.08.002 28969789

[B19] Huang Y. Y. He C. T. Shen C. Guo J. J. Mubeen S. Yuan J. G.. (2017). Toxicity of Cadmium and its health risks from leafy vegetable consumption. Food Funct. 8, 1373–1401. doi: 10.1039/C6FO01580H 28232985

[B20] Huh W. K. Falvo J. V. Gerke L. C. Carroll A. S. Howson R. W. Weissman J. S.. (2003). Global analysis of protein localization in budding yeast. Nature 425, 686–691. doi: 10.1038/nature02026 14562095

[B21] Jarup L. Akesson A. (2009). Current status of Cadmium as an environmental health problem. Toxicol. Appl. Pharmacol. 238, 201–208. doi: 10.1016/j.taap.2009.04.020 19409405

[B22] Jin Z. M. Deng S. Q. Wen Y. C. Jin Y. F. Pan L. Zhang Y. F.. (2019). Application of Simplicillium chinense for Cd and Pb biosorption and enhancing heavy metal phytoremediation of soils. Sci. Total Environ. 697, 134148. doi: 10.1016/j.scitotenv.2019.134148 31479903

[B23] Jin H. L. Martin C. (1999). Multifunctionality and diversity within the plant MYB-gene family. Plant Mol. Biol. 41, 577–585. doi: 10.1023/A:1006319732410 10645718

[B24] Kamran M. Malik Z. Parveen A. Zong Y. T. Abbasi G. H. Rafiq M. T.. (2019). et al: Biochar alleviates Cd phytotoxicity by minimizing bioavailability and oxidative stress in pak choi (*Brassica chinensis* L.) cultivated in Cd-polluted soil. J. Environ. Manage 250, 109500. doi: 10.1016/j.jenvman.2019.109500 31513996

[B25] Katiyar A. Smita S. Lenka S. K. Rajwanshi R. Chinnusamy V. Bansal K. C. (2012). Genome-wide classification and expression analysis of MYB transcription factor families in rice and Arabidopsis. BMC Genomics 13, 544. doi: 10.1186/1471-2164-13-544 23050870 PMC3542171

[B26] Kim S. H. Kim H. S. Bahk S. An J. Yoo Y. Kim J. Y.. (2017). Phosphorylation of the transcriptional repressor MYB15 by mitogen-activated protein kinase 6 is required for freezing tolerance in Arabidopsis. Nucleic Acids Res. 45, 6613–6627. doi: 10.1093/nar/gkx417 28510716 PMC5499865

[B27] Kuboi T. Noguchi A. Yazaki J. (1986). Family-dependent cadmium accumulation characteristics in higher-plants. Plant Soil 92, 405–415. doi: 10.1007/BF02372488

[B28] Lee J. S. Chon H. T. Kim K. W. (2005). Human risk assessment of As, Cd, Cu and Zn in the abandoned metal mine site. Environ. Geochemistry Health 27, 185–191. doi: 10.1007/s10653-005-0131-6 16003586

[B29] Li Z. L. Liu Z. H. Chen R. J. Li X. J. Tai P. D. Gong Z. Q.. (2015). DNA damage and genetic methylation changes caused by Cd in Arabidopsis thaliana seedlings. Environ. Toxicol. Chem. 34, 2095–2103. doi: 10.1002/etc.3033 25914311

[B30] Lin C. R. Lee K. W. Chen C. Y. Hong Y. F. Chen J. L. Lu C. A.. (2014). SnRK1A-interacting negative regulators modulate the nutrient starvation signaling sensor SnRK1 in source-sink communication in cereal seedlings under abiotic stress. Plant Cell 26, 808–827. doi: 10.1105/tpc.113.121939 24569770 PMC3967042

[B31] Liu J. Y. Osbourn A. Ma P. D. (2015). MYB transcription factors as regulators of phenylpropanoid metabolism in plants. Mol. Plant 8, 689–708. doi: 10.1016/j.molp.2015.03.012 25840349

[B32] Liu C. Y. Xie T. Chen C. J. Luan A. P. Long J. M. Li C. H.. (2017). Genome-wide organization and expression profiling of the R2R3-MYB transcription factor family in pineapple (*Ananas comosus*). BMC Genomics 18 (1). doi: 10.1186/s12864-017-3896-y PMC5494133 28668094

[B33] Melila M. Rajendran R. Lumo A. K. Arumugam G. Kpemissi M. Sadikou A.. (2019). Cardiovascular dysfunction and oxidative stress following human contamination by fluoride along with environmental xenobiotics (Cd & Pb) in the phosphate treatment area of Togo, West Africa. J. Trace Elements Med. Biol. 56, 13–20. doi: 10.1016/j.jtemb.2019.07.002 31442949

[B34] Parrotta L. Guerriero G. Sergeant K. Cal G. Hausman J. F. (2015). Target or barrier? The cell wall of early- and later-diverging plants vs cadmium toxicity: differences in the response mechanisms. Front. Plant Sci. 6. doi: 10.3389/fpls.2015.00133 PMC4357295 25814996

[B35] Rizwan M. Ali S. Adrees M. Ibrahim M. Tsang D. C. W. Zia-Ur-Rehman M.. (2017). A critical review on effects, tolerance mechanisms and management of Cadmium in vegetables. Chemosphere 182, 90–105. doi: 10.1016/j.chemosphere.2017.05.013 28494365

[B36] Salih H. Gong W. F. He S. P. Sun G. F. Sun J. L. Du X. M. (2016). Genome-wide characterization and expression analysis of MYB transcription factors in Gossypium hirsutum. BMC Genet. 17 (1), 129. doi: 10.1186/s12863-016-0436-8 27613381 PMC5017022

[B37] Sapara K. K. Khedia J. Agarwal P. Gangapur D. R. Agarwal P. K. (2019). SbMYB15 transcription factor mitigates Cadmium and nickel stress in transgenic tobacco by limiting uptake and modulating antioxidative defence system. Funct. Plant Biol. 46, 702–714. doi: 10.1071/FP18234 31023418

[B38] Sasaki A. Yamaji N. Yokosho K. Ma J. F. (2012). Nramp5 is a major transporter responsible for manganese and cadmium uptake in rice. Plant Cell 24, 2155–2167. doi: 10.1105/tpc.112.096925 22589467 PMC3442593

[B39] Ueno D. Yamaji N. Kono I. Huang C. F. Ando T. Yano M.. (2010). Gene limiting cadmium accumulation in rice. Proc. Natl. Acad. Sci. United States America 107, 16500–16505. doi: 10.1073/pnas.1005396107 PMC2944702 20823253

[B40] Wang X. P. Niu Y. L. Zheng Y. (2021). Multiple functions of MYB transcription factors in abiotic stress responses. Int. J. Mol. Sci. 22 (11), 6125. doi: 10.3390/ijms22116125 34200125 PMC8201141

[B41] Yan C. Melese T. (1993). Multiple regions of NSR1 are sufficient for accumulation of a fusion protein within the nucleolus. J. Cell Biol. 123, 1081–1091. doi: 10.1083/jcb.123.5.1081 8245119 PMC2119886

[B42] Yang D. P. Guo Z. Q. Green I. D. Xie D. T. (2016). Effect of cadmium accumulation on mineral nutrient levels in vegetable crops: potential implications for human health. Environ. Sci. pollut. Res. 23, 19744–19753. doi: 10.1007/s11356-016-7186-z 27411535

[B43] Yang Y. Zhang F. S. Li H. F. Jiang R. F. (2009). Accumulation of Cadmium in the edible parts of six vegetable species grown in Cd-contaminated soils. J. Environ. Manage 90, 1117–1122. doi: 10.1016/j.jenvman.2008.05.004 18583020

[B44] Yang L. P. Zhu J. Wang P. Zeng J. Tan R. Yang Y. Z.. (2018). Effect of Cd on growth, physiological response, Cd subcellular distribution and chemical forms of Koelreuteria paniculata. Ecotoxicology Environ. Saf. 160, 10–18. doi: 10.1016/j.ecoenv.2018.05.026 29783107

[B45] Zhang H. H. Li X. Xu Z. S. Wang Y. Teng Z. Y. An M. J.. (2020). Toxic effects of heavy metals Pb and Cd on mulberry (Morus alba L.) seedling leaves: Photosynthetic function and reactive oxygen species (ROS) metabolism responses. Ecotoxicology Environ. Saf. 195, 110469. doi: 10.1016/j.ecoenv.2020.110469 32179235

[B46] Zhang P. Wang R. L. Ju Q. Li W. Q. Tran L. S. P. Xu J. (2019). The R2R3-MYB transcription factor MYB49 regulates cadmium accumulation. Plant Physiol. 180, 529–542. doi: 10.1104/pp.18.01380 30782964 PMC6501104

[B47] Zhao K. Cheng Z. H. Guo Q. Yao W. J. Liu H. J. Zhou B. R.. (2020). Characterization of the poplar R2R3-MYB gene family and over-expression of PsnMYB108 confers salt tolerance in transgenic tobacco. Front. Plant Sci. 11. doi: 10.3389/fpls.2020.571881 PMC7596293 33178243

[B48] Zhou Y. Y. Zhu H. He SZ Zhai H. Zhao N. Xing SH. (2019). A novel sweetpotato transcription factor gene IbMYB116 enhances drought tolerance in transgenic Arabidopsis. Front. Plant Sci. 10. doi: 10.3389/fpls.2019.01025 PMC6704235 31475022

[B49] Zhu S. J. Shi W. J. Jie Y. C. Zhou Q. M. Song C. B. (2020). A MYB transcription factor, BnMYB2, cloned from ramie (Boehmeria nivea) is involved in cadmium tolerance and accumulation. PLoS One 15. doi: 10.1371/journal.pone.0233375 PMC7233596 32421756

