# Corrigendum: BrMYB116 transcription factor enhances Cd stress tolerance by activating FIT3 in yeast and Chinese cabbage

**DOI:** 10.3389/fpls.2024.1462501

**Published:** 2024-08-14

**Authors:** Ali Anwar, Chao Yuan, Bing Cui, Lixia Wang, Lilong He, Jianwei Gao

**Affiliations:** ^1^ Institute of Vegetables, Shandong Key Laboratory of Greenhouse Vegetable Biology, Shandong Branch of National Vegetable Improvement Center, Huanghuai Region Vegetable Scientific Station of Ministry of Agriculture (Shandong), Shandong Academy of Agricultural Sciences, Jinan, China; ^2^ College of Horticulture, South China Agriculture University, Guangzhou, China; ^3^ Key Laboratory of Plant Development and Environment Adaptation Biology, Ministry of Education; School of Life Science, Shandong University, Qingdao, China

**Keywords:** Chinese cabbage, abiotic stress, BrMYB116, FIT3, Cd stress, RNA-Seq

In the published article, there was an error in **Figure 8** as published. The corrected **Figure 8** and its caption appear below.

**Figure 8:**
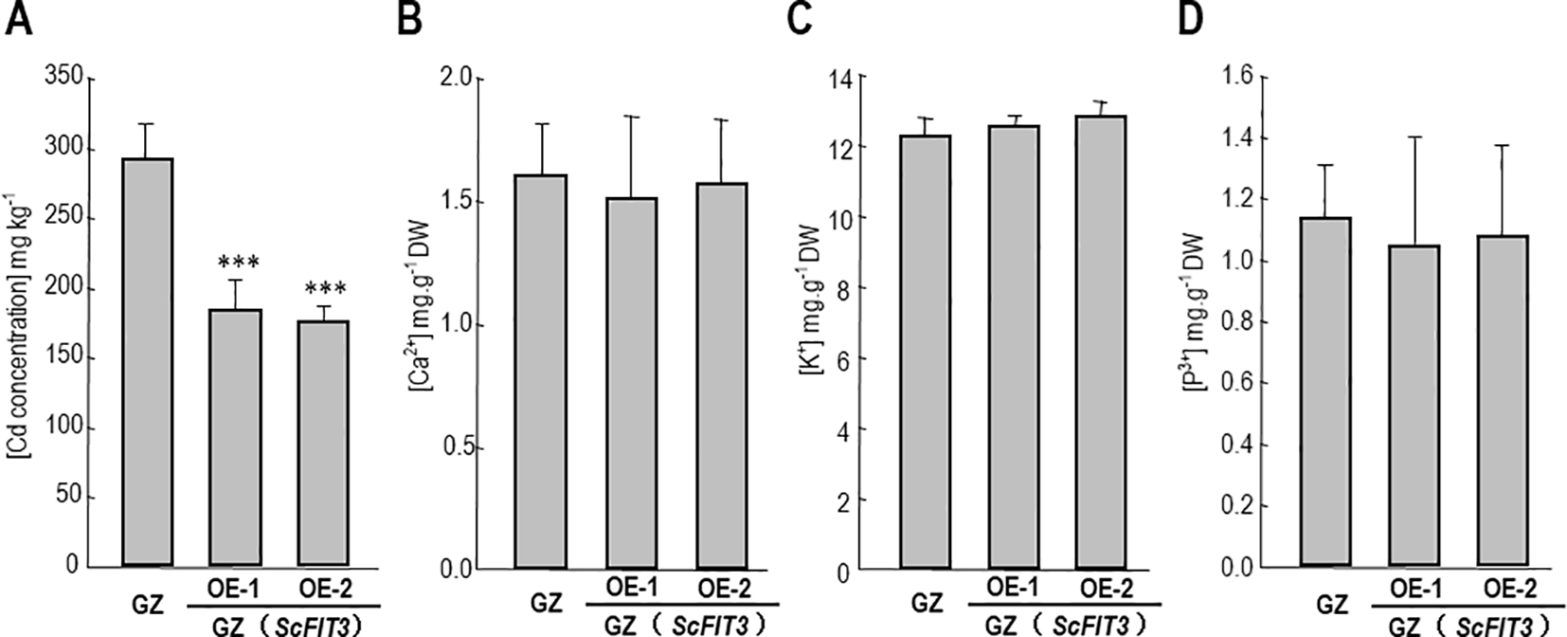
Cd content and some nutrition ion contents in the Chinese cabbage. The content of Cd **(A)**, Ca^2+^ **(B)**, K^+^ **(C)**, p^3+^ **(D)** in the wild-type and ScFIT3 transgenic Chinese cabbage. Error bars indicate ± SD of three biological repeats. P value of student’s t test: BrMYB116 or ScFIT3 transgenic plants compared with the wild-type control. ***P<0.001.

The authors apologize for this error and state that this does not change the scientific conclusions of the article in any way. The original article has been updated.

