# Supplementary material for: BrMYB116 transcription factor enhances Cd stress tolerance by activating FIT3 in yeast and Chinese cabbage
Source: Front Plant Sci. 2024 Jun 7;15:1388924. doi: 10.3389/fpls.2024.1388924 (PMC11190832; doi:10.3389/fpls.2024.1388924)
Supplement: Supplementary file 1 [file DataSheet1.zip › Supplementary Figures S1, S2 and S3.pdf]

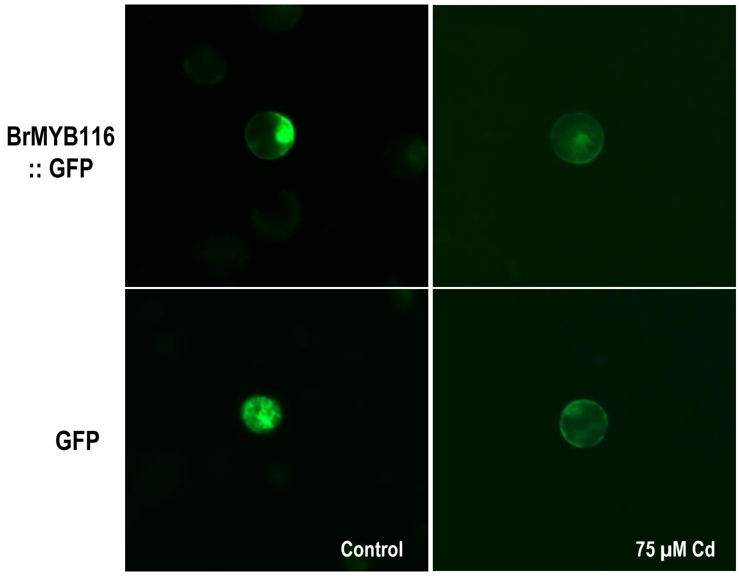

Supplementary Figure 1. Subcellular localization of BrMYB116 fused to GFP and transiently expressed in Arabidopsis protoplasts treated without or with Cd (75 μM $CdCl_2$, 2 h).

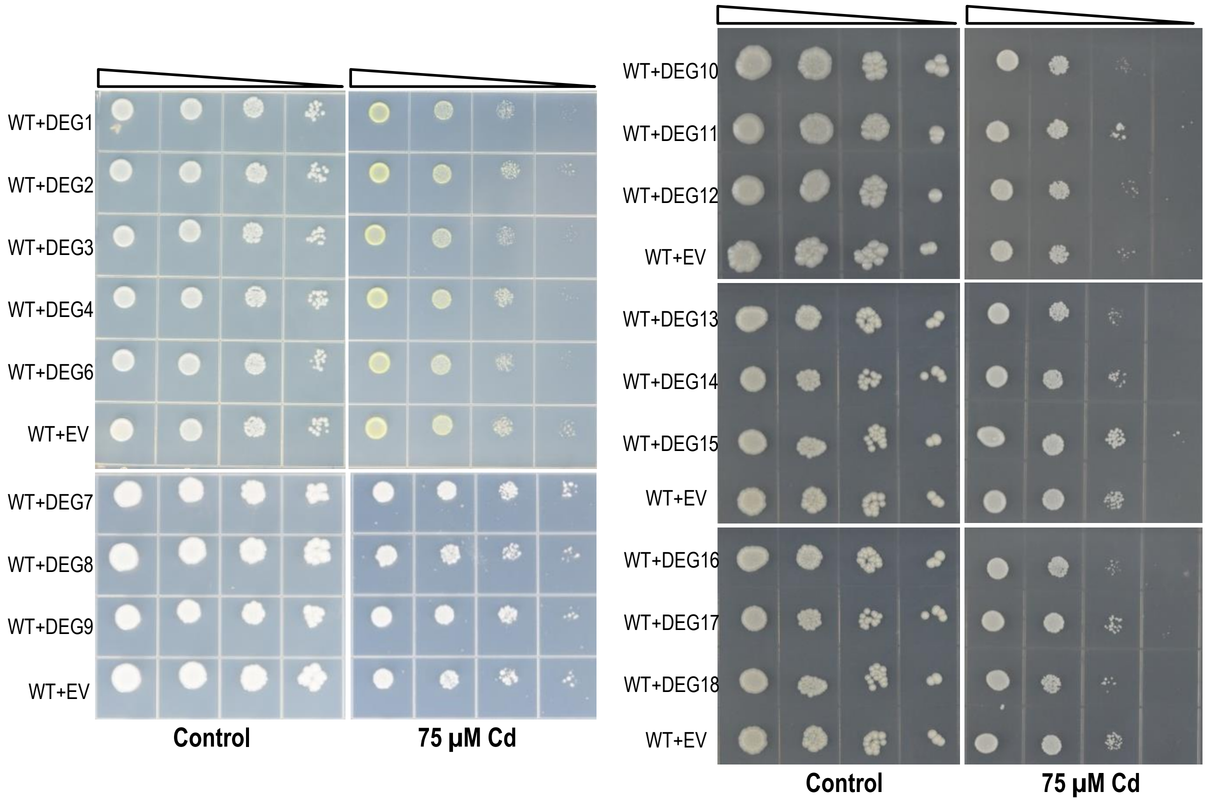

Supplementary Figure 2. Dilution bioassay for the wild-type yeast strain and the wild-type strain overexpressing 17 DEGs in the SC medium. Triangles represent serial 10-fold dilutions (starting concentration of 0.3 OD_{600}). The representative test from three reproducible experiments is shown.

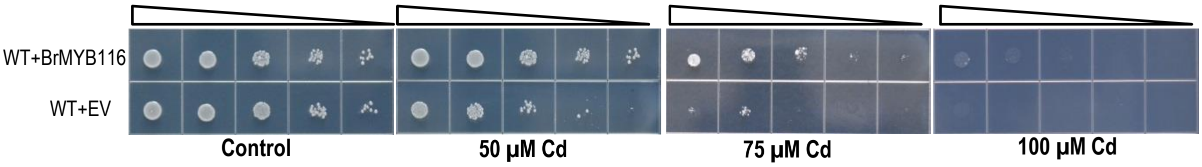

Supplementary Figure 3. Dilution bioassay for the wild-type strain overexpressing BrMYB116 and empty vector in the SC medium. Triangles represent serial 10-fold dilutions (starting concentration of 0.3 OD_{600}). The representative test from three reproducible experiments is shown.
