# Supplementary material for: BrMYB116 transcription factor enhances Cd stress tolerance by activating FIT3 in yeast and Chinese cabbage
Source: Front Plant Sci. 2024 Jun 7;15:1388924. doi: 10.3389/fpls.2024.1388924 (PMC11190832; doi:10.3389/fpls.2024.1388924)
Supplement: Supplementary file 1 [file DataSheet1.zip › Supplementary TableS1 and S3.pdf]

Supplemental Table S1. Primer Sequences. Capital lettering indicates sequences from genes, lower case from vectors.

Primer name	Sequence
BraMYB116 (CDS) F	5' ATGTCAAATATAACAAAGAA 3'
BraMYB116 (CDS) R	5' CTAATTAAAGTGTGCATACT 3'
ScFIT3 (CDS) F	5' ATGAAATTCTCTTCCGCTTTGGTTC 3'
ScFIT3 (CDS) R	5' TTACAATAACATGACGGCAGCAAG 3'
BrMYB116-pRS416-GFP F	5' caaatctagaactagATGTCAAATATAATGAAGAAGAGGTGTAATGAAAAT 3'
BrMYB116-pRS416-GFP R	5' cgggggatccactagaATTAAAAGGTGCGTACTCCTCAAAC 3'
ScFIT3-pRS416-GFP F	5' caaatctagaactagATGAAATTCTCTTCCGCTTTGGT 3'
ScFIT3-pRS416-GFP R	5' cgggggatccactaggCAATAACATGACGGCAGCAAGG 3'
NSR1 IF423 F(BamH1)	5' gctctagaactagtgATGGCTAAGACTACTAAAGTAAAAG 3'
NSR1 IF423 R(BamH1)	5' GTCCTCGGTGTTGTCTCCTCCTCCTCCTCCTCCATCAAATGTTTTCTTTGAACC 3'
VLD1 IF423 F(BamH1）	5' ATGTTGCATTTGGAAGATGAC 3'
VLD1 IF423 R(BamH1）	5' GTCCTCGGTGTTGTCTCCTCCTCCTCCTCCTTCGGCGATATTCAGTAGAGG 3'
DsRFP IF423 F(BamH1）	5' GACAACACCGAGGACGTCATC 3'
DsRFP IF423 R(BamH1）	5' gcagcccgggggatcCTACTGGGAGCCGGAGTG 3'
BrMYB116 IF3300 F(Xba1)	5' gtacccggggatcctTGATAGCCACTGATAGGTTT 3'
BrMYB116 IF3300 F(Xba1)	5' gcaggtcgactctagCTAATTAAAAGGTGCGTACT 3'
ScFIT3 IF3300 F(Xba1)	5' gtacccggggatcct TTAGCCCATTTTTCACTGTT 3'
ScFIT3 IF3300 F(Xba1)	5' gcaggtcgactctagTTACAATAACATGACGGCA 3'
**Luciferase assay constructs**	
Firefly luciferase F	5' CCCTAAACCCTAAAATGGAAGACGCCAAA 3'
Firefly luciferase R	5' CGACTCTAGAGGATCCGACTCTAGAGGATCTTACACGG 3'
Fluc-pCambia3300 F	5' CCCTAAAATGGAAGACGCCAAAAACATAAAGAA 3'
Fluc-pCambia3300 R	5' CGACTCTAGAGGATCTTACACGGCGATCTTTCCGC 3'
pScFIT3 F	5' TTTAGGGATTATTGTTATTAGTTATTTTT 3'
pScFIT3 R	5' TTAGCCCATTTTTCACTG 3'
pScFIT3-pCambia3300 F	5' gaattcccggggatcTTTAGGGATTATTGTTATTAGTTATTTTTAAGCGAGTGG 3'
pScFIT3-pCambia3300 R	5' gcaggtcgacggatcTTAGCCCATTTTTCACTGTTATCACAACT 3'
**RT-qPCR primers**	
BraMYB116 q F	5' TGGACACTCGAGGAAGACAC 3'
BraMYB116 q R	5' CCACGCTTTATGTCGGGTTT 3'
BrACT2 q F	5’ GAGATTCACATGCCCAGAAGTCTTG 3’
BrACT2 q R	5’ ACCTGCCTCATCATACTCGGC 3’

Supplementary Table S3. Significantly enriched Gene Ontology (GO) terms of DEGs. Expression patterns of some DEGs annotated to the given GO-term. Corrected P-value ≤ 0.05 defined as significantly enriched GO terms in DEGs.

Gene Ontology term	Cluster frequency	Corrected P Value
transporter activity	20 out of 155 genes, 14.2%	0.020405095
transmembrane transporter activity	17 out of 155 genes, 11.0%	0.020405095
