# Supplementary figures and images for: BrMYB116 transcription factor enhances Cd stress tolerance by activating FIT3 in yeast and Chinese cabbage
Source: Front Plant Sci. 2024 Jun 7;15:1388924. doi: 10.3389/fpls.2024.1388924 (PMC11190832; doi:10.3389/fpls.2024.1388924)

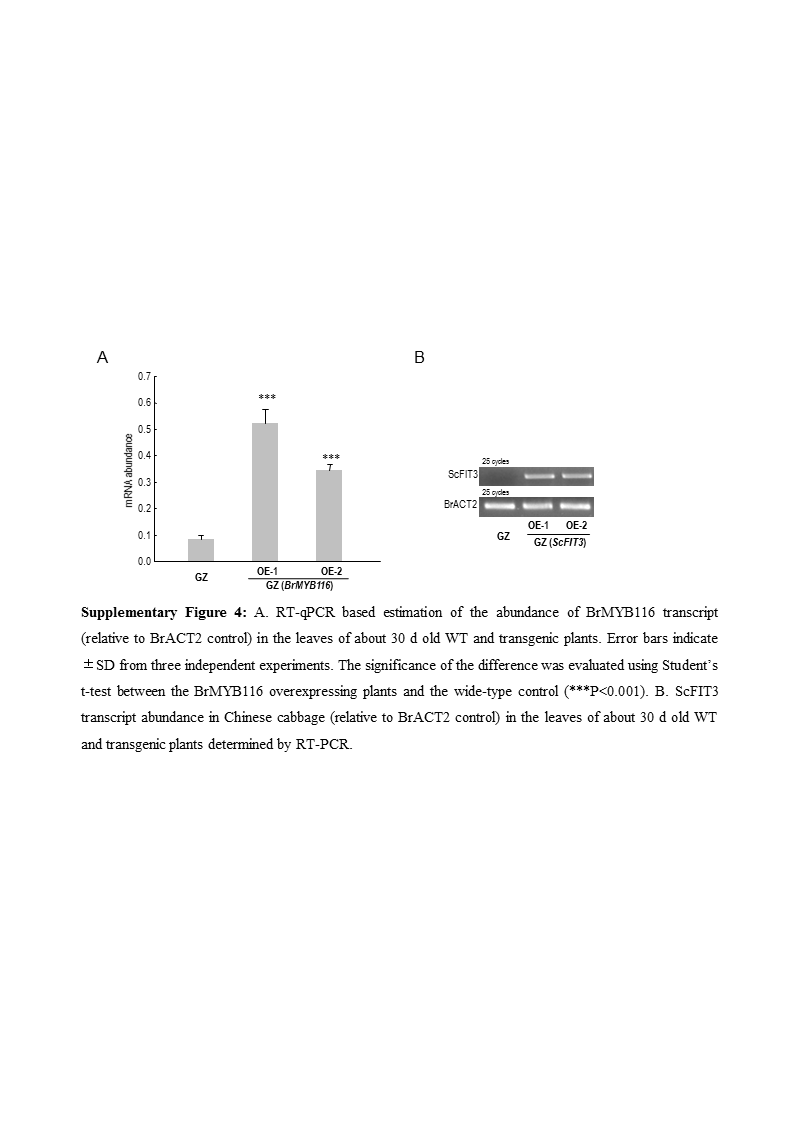

Supplement: Supplementary file 1 [file DataSheet1.zip › Supplementary Figure S4.TIF]
